# Efficacy of novel osteotomy techniques in the treatment of congenital radial head dislocation

**DOI:** 10.3389/fped.2026.1683592

**Published:** 2026-02-04

**Authors:** Wusheng Miao, Bo Li, Junjun Li, Kun Zhang, Xiaobo Zhang

**Affiliations:** 1 Department of Pediatric Orthopedics, The Third Affiliated Hospital of Xi'an Medical University, Shaanxi, China; 2 Department of Orthopedics, Honghui Hospital, Xi'an Jiao Tong University, Shaanxi, China

**Keywords:** clinical efficacy, congenital radial head dislocation, double osteotomy, posterior shift osteotomy, radiographic outcomes, rotational osteotomy

## Abstract

**Objective:**

To evaluate the clinical curative effect and imaging results of osteotomy and rotation surgery for congenital radial head dislocation.

**Methods:**

A retrospective analysis was conducted on 20 children with congenital radial head dislocation who underwent clinical surgical treatment meeting the inclusion criteria from June 2,015 to June 2024 via osteotomy and rotation surgery. The sex, age, and follow-up time of the children were recorded. Clinical efficacy was assessed through elbow joint range of motion (pronation, supination, flexion), and imaging results were evaluated via the Nakamura grading system, while related complications such as wound infection and neuronal injury were recorded.

**Results:**

All surgeries were completed, with 14 males and 6 females aged 3–15 years, with an average age of 7.05 ± 3.98 years, and a follow-up time of 12–120 months, with an average of 33.95 ± 37.65 months. X-ray images of the elbow joint revealed that the humeroradial joint was reduced, with no recurrence of radial head dislocation, no nonunion at the osteotomy site, and no developmental abnormalities of the proximal ulna. The preoperative elbow joint pronation angle was 81.15° ± 3.65°, supination angle was 83.15° ± 3.70°, and flexion angle was 129.80° ± 4.56°; the postoperative elbow joint pronation angle was 80.30° ± 4.44°, supination angle was 84.75° ± 3.24°, and flexion angle was 138.25° ± 3.46°. Among them, the hyperextension state of one child with elbow hyperextension disappeared after surgery. The range of motion of the elbow joint improved significantly. Nakamura grading revealed that 85.0% (17/20) of the children were excellent, and 15.0% (3/20) were good. No wound infections or neuronal injuries occurred in any of the children after surgery.

**Conclusion:**

Proximal ulnar osteotomy and rotation surgery is an effective treatment method for congenital radial head dislocation, demonstrating significant clinical efficacy and safety.

## Introduction

Congenital radial head dislocation (CRHD) is a rare congenital abnormality that is usually discovered during childhood (1, 2), with an incidence rate ranging from 0.06% to 0.16% (3–5). Approximately 60% of children with congenital radial head dislocation have other congenital upper limb abnormalities (6, 7). The etiology of congenital radial head dislocation is currently unclear and may be related to other genetic diseases or the stage of embryo development (1, 8, 9). Early on, there are usually no clinical symptoms, and it is often discovered because of a mass in front of the elbow joint, limited elbow joint movement, or x-ray examination after trauma; a few adolescent patients may exhibit mild pain, clicking sounds, or limited flexion of the elbow joint (10). Although there are various treatment methods, including open reduction and annular ligament reconstruction, scholars have not yet reached a consensus on the pathological changes associated with CRHD due to the complexity and diversity of the disease.

In recent years, with the advancement of medical technology, osteotomy rotation has gradually attracted academic attention as an effective surgical technique. These methods aim to improve the range of motion and function of the elbow joint, alleviate patient pain, and restore the normal anatomical structure of the elbow joint through different surgical approaches and techniques.

## Methods

### Basic information

A retrospective analysis was conducted on 20 child patients with CRHD who underwent surgical treatment from April 2011 to June 2024, with an average age of 7.05 years (ranging from 3 to 15 years), including 14 males and 6 females. All surgeries were performed by the first author to ensure consistency in surgical technique. The inclusion criteria were a clear diagnosis of CRHD; patients aged between 3 and 15 years; no other severe diseases affecting elbow function; no clear history of trauma; x-ray showing anterior dislocation of the radial head; and preoperative MRI showing an intact proximal radioulnar joint, no scar tissue in the elbow joint, and the main pathological change was abnormal positioning of the proximal radioulnar joint, located anterolaterally instead of laterally. The exclusion criteria included previous surgical treatment for radial head dislocation; other upper limb skeletal deformities; not meeting the inclusion criteria for x-ray or MRI; dislocation of the proximal radioulnar joint; old fracture of the upper end of the ulna complicated with dislocation of the radial head; and loss or incompleteness of follow-up data.

### Surgical methods

The operation adopted the Boyd approach to the posterior lateral elbow joint. After the elbow joint was exposed, we first observed whether the humeroradial, humeroulnar, and radioulnar joints were within a single joint cavity and then observed the position of the ulnar notch and the stability of the radioulnar joint. The integrity of the annular ligament was checked, and the osteotomy plane of the ulna, which is located 2 mm below the coronoid process of the ulna and is as parallel as possible to the articular surface of the radial head, was determined. After osteotomy of the ulna, the upper radioulnar joint (complex) is rotated as a whole posteriorly and laterally, with the degree of rotation (approximately 30°–50°) determined by the intraoperative goal of restoring optimal congruence of the humeroradial joint rather than a predetermined angle.

The restoration of humeroradial congruency was assessed intraoperatively through a combination of direct visualization and dynamic fluoroscopic evaluation. After rotating the complex, the articular alignment was directly inspected. Subsequently, under C-arm fluoroscopy, the elbow was passively taken through a range of motion (flexion-extension and pronation-supination). A congruent reduction was confirmed by the absence of subluxation and the maintenance of a symmetric joint space throughout this dynamic assessment on anteroposterior, lateral, and oblique views.

**Figure 1:**
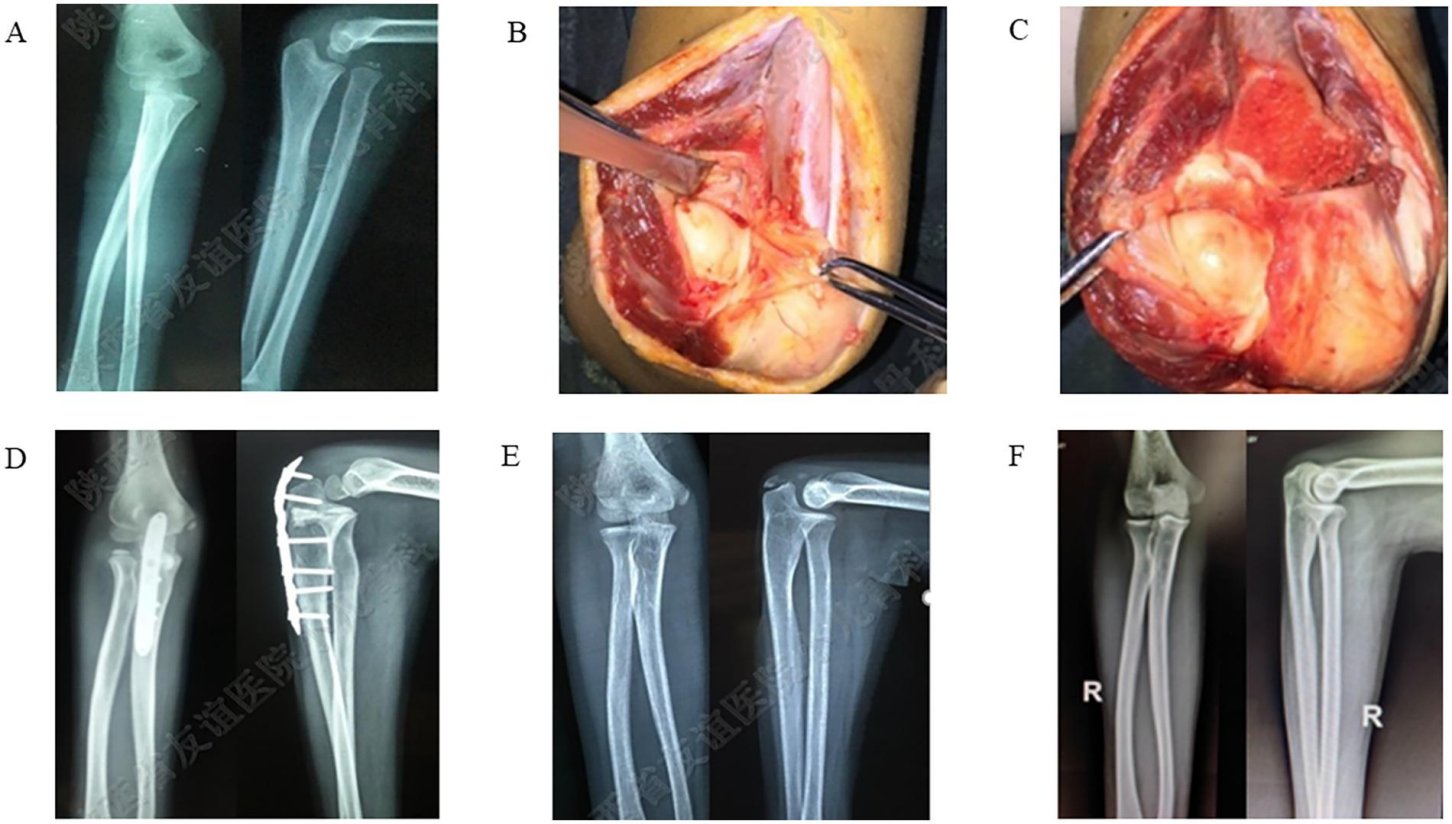
A 7-year-old male patient with a congenitally dislocated radius head on the right side. **(A)** Preoperative elbow x-ray shows anterior dislocation of the radial head; **(B,C)** Intraoperative photographs reveal proximal overlap of the ulna and radius, cubitus valgus deformity, and intact radioulnar joint. **(D)** Immediate postoperative DR demonstrates a well-positioned radial head, with normal radioulnar and humeroradial relationships. **(E,F)** DR images at 3 years and 10 years postoperatively show no dislocation of the radial head, normal elbow joint relationships.

**Figure 2:**
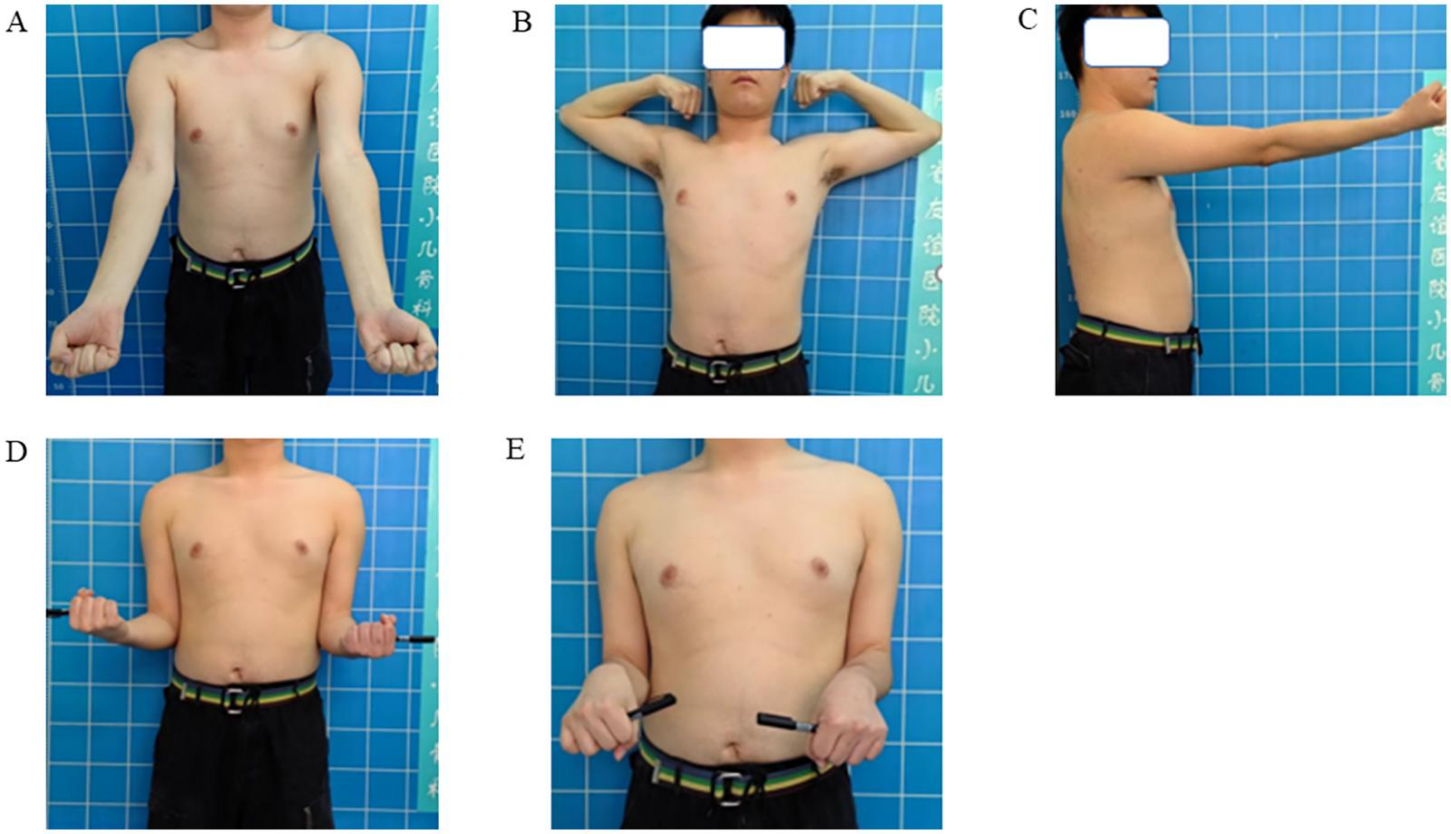
Macroscopic photographs at the 10-year follow-up after surgery, and normal range of motion of the elbow joint. **(A)** Elbow extension view; **(B)** shoulder abduction with elbow flexion position; **(C)** elbow extension lateral view; **(D)** supine position; **(E)** prone position.

After the radial head is reduced, 1.5 mm K-wires were used initially for cross fixation of the osteotomy site in all cases to provide rotational stability. The decision for definitive fixation was then made based on an intraoperative assessment of bony stability. In the majority of cases, a locking compression plate (LCP) was added. In two cases involving older patients with excellent bone quality and exceptionally stable osteotomy contact, plate fixation alone was deemed sufficient. The stability of the humeroradial joint during flexion and extension of the elbow joint and rotation of the forearm is observed. Intraoperatively, fluoroscopic positioning is performed to ensure that the plate and screws do not damage the epiphysis, confirming that the radial head has been reduced. After the operation, the elbow joint was flexed to 90°, and the forearm was in the supinated position, with the upper limb fixed in a plaster cast for 3–4 weeks before elbow joint functional exercises were started. The internal fixation hardware was typically removed approximately 8 months postoperatively, depending on radiographic evidence of union. Typical cases are shown in Figures 1, 2. Surgical demonstration diagram is shown in Supplementary Document S1.

### Clinical indicators and radiographic indices

Clinical indicators are assessed through the range of motion of the elbow joint (pronation, supination, elbow flexion, and elbow extension). The Nakamura grading system is used to evaluate imaging findings and the degree of functional recovery: excellent is complete reduction, asymptomatic, and normal function; good is basic reduction, mild symptoms, and function close to normal; and poor is unreduced or redislocated, significant symptoms, and severely limited function.

### Complications

The incidence of complications such as wound infection, neuronal injury, and nonunion or delayed healing at the osteotomy site was recorded.

### Data statistics and analysis

All the data were statistically analyzed via SPSS 26.0 software, and the continuous variables are expressed as the means ± standard deviations.

## Results

All the children completed the surgery, with no wound infections or neuronal injuries postoperatively. Bone healing at the osteotomy site occurred within 3–6 months. The mean age was 7.05 ± 3.98 years, with a sex distribution of 14 males and 6 females. The follow-up period ranged from 12 to 108 months, with an average of 33.95 ± 37.65 months, as shown in Table 1. X-ray images of the elbow joint indicated reduction of the humeroradial joint, with no recurrence of the dislocated radius head, and the osteotomy site had healed within 3–6 months post-operatively, with no abnormalities in the development of the proximal ulna.

**Table 1:** Basic information.

Category	Number
Patient number	20
Sex (male/female)	14/6
Affected side(left/right)	5/6
Mean age	7.05 ± 3.98 years
Follow-up duration	33.95 ± 37.65 months

### Elbow joint range of motion

The average pronation angle of the elbow joint was 81.15° ± 3.65°, and the average supination angle was 83.15° ± 3.70°. There was one child with elbow hyperextension. The average elbow flexion angle was 129.80° ± 4.56°, as shown in Table 2; the postoperative elbow pronation angle was 80.30° ± 4.44°; the postoperative elbow supination angle was 84.75° ± 3.24°; and hyperextension in the child with elbow hyperextension disappeared. The average postoperative elbow flexion angle was 138.25° ± 3.46°, as shown in Table 2.

**Table 2:** Range of motion of the elbow joint in children before and after surgery.

Group	Preoperative	Postoperative	*F*	*P*
Pronation	81.15 ± 3.65	80.30 ± 4.44	2.135	0.512
Supination	83.15 ± 3.70	84.75 ± 3.24	0.717	0.154
Elbow flexion	129.80 ± 4.56	138.25 ± 3.46	1.263	**<0**.**001**

*P* < 0.05 is indicated in bold.

All 20 patients had varying degrees of flexion and extension activity limitations preoperatively, and 18 patients experienced significant improvement in elbow flexion and extension range of motion postoperatively, 1 patient experienced no significant change, and 1 patient experienced limited extension activity. Seventeen patients had normal forearm rotation preoperatively, 1 patient had limited supination, and 2 patients had limited pronation. There was no significant change in pronation or supination activity postoperatively compared with preoperatively. The pain and daily activities of the elbow joint did not significantly change preoperatively or postoperatively.

### Nakamura classification

The Nakamura classification revealed that 85.0% (17/20) of the children were excellent, and 15.0% (3/20) of the children were good. All the children had no wound infection or nerve injury after surgery.

## Discussion

Children with congenital radial head dislocation (CRHD) are often asymptomatic in the early stages and experience mild pain and limited flexion and extension of the elbow joint before puberty. As they age, pain and elbow joint dysfunction gradually worsen. Imaging findings include a relatively short ulna and radius or long radius; hypoplasia or absence of the humeral head; partial absence of the trochlea; a long and thin radial neck; a rounded radial head; a prominent medial condyle of the humerus; and ulnar bowing, with deformities becoming more pronounced as the child grows older.

Thus far, surgical treatment remains a clinical challenge. Surgical methods are complex and varied and include open reduction osteotomy of the ulna, open reduction of the ulna with annular ligament reconstruction, and radial head resection. Some scholars have reported the effectiveness of proximal radial resection and partial excision of the anterior joint capsule of the elbow joint (11). Tsumura *et al*. (12) combined radial osteotomy with a custom device, ulnar osteotomy, and local adipofascial flap elevation, resulting in significant improvement in the range of motion of the elbow joint postoperatively. Radial head resection is a classic technique that can increase the range of motion and reduce elbow pain (13), but it may lead to complications such as decreased elbow joint stability, limited forearm rotation function, and elbow joint pain after resection. Some scholars have used open reduction of the ulnar osteotomy and annular ligament reconstruction to achieve radial head reduction, with complications such as laxity, contracture, or rerupture of the annular ligament, limited forearm rotation function, and postoperative redislocation (10). After ulnar osteotomy, expansion and loosening of the annular ligament can achieve radial head reduction (14), and annular ligament reconstruction is not mandatory (15). It has been reported that for children with CRHD, subsequent surgical correction after osteotomy does not provide significant benefits in cases of redislocation (16).

This study retrospectively analyzed and compared the clinical efficacy and imaging effects of osteotomy rotation in the treatment of CRHD. A total of 20 children were included in this study, with an average follow-up time of more than 24 months, during which no long-term complications were observed. In terms of joint range of motion (pronation, supination, elbow flexion, and elbow extension), postoperative flexion and extension activities increased compared with those before surgery, whereas there was no significant change in pronation and supination activities postoperatively compared with those before surgery. Notably, even with a derotation of the ulna estimated up to 50° in some cases, forearm rotation was preserved. This can be explained by the distinct anatomy and biomechanics of the forearm. Pronation and supination primarily occur at the distal and proximal radioulnar joints. The osteotomy is performed on the proximal ulna to correct the translational dislocation of the radial head relative to the capitellum. Derotating the ulna changes the spatial position of the radial head to reduce it, but does not alter the axial relationship for the radius rotating around the ulna. Once the reduced proximal radioulnar joint relationship is restored, the arc of radial rotation is maintained. Thus, the surgery addresses the malposition of the radial head without compromising the fundamental mechanism of forearm rotation. The Nakamura imaging grading system showed good imaging clinical efficacy. This study demonstrated that proximal ulnar osteotomy rotation can effectively restore the normal anatomical structure and function of the elbow joint, with no occurrence of nerve injury, infection, or other complications and no recurrence of dislocation during the follow-up period, providing new possibilities for the treatment of CRHD. Our research results indicate that 85% of patients were rated as excellent according to the Nakamura grading system, further confirming the efficacy of this surgical plan.

The ulnar annular ligament and the ulnar radial notch form a fibrous bone ring to accommodate the rotation of the radius. This study adjusted the ulnar radial notch through ulnar rotational osteotomy, which involves rotating the ulna to adjust the position of the radial head and restore its normal anatomical relationship with the humerus. This surgical method is suitable for patients with radial head dislocation, stable radioulnar joints, and deformities of the ulnar radial notch directed anteriorly. Its advantages include relatively little surgical trauma, a shorter recovery time, and significant improvement in the rotational activity of the elbow joint. Osteotomy rotation involves osteotomy of the upper end of the ulna, with the distal end rotating outward and backward, restoring the ulnar radial notch to its normal lateral position, allowing for the reduction of the radial head, and the ulna does not need to be angled backward, which has little impact on elbow extension activities. If the osteotomy plane is below the upper radioulnar joint surface, it will damage the existing radioulnar joint. In our previous study, osteotomy rotation was shown to be an effective method for treating CRHD (17). Research has shown through three-dimensional CT that children with old radial head dislocation have three-dimensional deformities of the ulna. They successfully treated radial head dislocation through ulnar rotational osteotomy (18, 19). The key to the success of this surgery is determining the plane of the ulnar osteotomy, which should be horizontal under the ulnar coronoid process. During osteotomy, care should be taken to protect the annular ligament from injury. Postoperatively, there may be issues of insufficient or excessive rotational angles, thus requiring high technical demands on the surgeon. During the procedure, accurate calculation of the rotational angle is essential to avoid postoperative limitations in elbow joint movement, and care must be taken to avoid damaging peripheral nerves and vascular structures.

## Conclusion

The rotational osteotomy method has shown significant clinical efficacy and safety in the treatment of CRHD. This study did not investigate cases of congenital posterior dislocation or lateral dislocation of the radial head, and the small sample size and limitations of a single-center study may lead to bias, affecting the generalizability of the results. Future multicenter, large-sample randomized controlled studies are needed to verify the effectiveness and safety of these surgical methods.

## Data Availability

The original contributions presented in the study are included in the article/Supplementary Material, further inquiries can be directed to the corresponding author.
