# Supplementary figures and images for: Efficacy of novel osteotomy techniques in the treatment of congenital radial head dislocation
Source: Front Pediatr. 2026 Feb 4;14:1683592. doi: 10.3389/fped.2026.1683592 (PMC12913493; doi:10.3389/fped.2026.1683592)

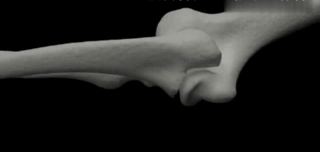


Supplementary Document 1

Supplement: Supplementary file 1 [file Supplementaryfile1.docx]
